# Leveraging Text Messaging and Behavior Theory to Improve Colorectal Cancer Screening in Federally Qualified Health Centers: Cohort Study

**DOI:** 10.2196/86408

**Published:** 2026-05-13

**Authors:** Tracy Angelocci, Tonghui Xu, Tushar Talaparthy, Cecilia Corral, Omolola E Adepoju

**Affiliations:** 1 Lone Star Circle of Care, Georgetown, TX, United States; 2 Humana Integrated Health Systems Science Institute, 5055 Medical Cir, Houston, TX, 77004, United States, 1 713-743-9706; 3 Tilman J. Fertitta Family College of Medicine, University of Houston, 5055 Medical Circle, Houston, TX, United States; 4 CareMessage, San Francisco, CA, United States

**Keywords:** colorectal cancer, cancer screening, primary care, social determinants of health, federally qualified health center, FQHC

## Abstract

**Background:**

Colorectal cancer (CRC) is the second leading cause of cancer-related deaths in the United States. Despite recommendations for screening to begin at the age of 45 years, significant disparities persist, particularly among medically underserved populations.

**Objective:**

This study examines the effectiveness of SMS text messaging reminders in improving CRC screening rates across 2 large federally qualified health centers (FQHCs) serving vulnerable populations.

**Methods:**

The study included 4822 adults aged ≥45 years, receiving care at 2 large FQHC networks in Texas and California. Participants were assigned to one of four groups: (1) control (no SMS text messages), (2) single-outreach SMS text overdue message, (3) three-week SMS overdue and reminder text messages, and (4) six-week SMS text messages that were informed by behavior theory. Data were collected from May 2023 to July 2024. The outcome measure was a binary indicator of whether the participant underwent 1 of 3 CRC tests, fecal immunochemical test, colonoscopy, and Cologuard, within 90 days of completing the SMS text messaging reminders. Independent variables included demographic, geographic, clinical, and primary care access variables. Multivariate logistic regression models were used to examine associations between CRC screening completion and the SMS text messaging reminder groups, adjusting for covariates. Adjusted odds ratios (aORs) and 95% CIs were reported.

**Results:**

In the combined 3-test model, patients in the single-outreach SMS text message (aOR 1.22, 95% CI 1.00-1.47) and the 3-week SMS text message (aOR 1.27, 95% CI 1.05-1.53) groups had higher odds of completing the screening test compared to those in the control group. Within the fecal immunochemical test–only model, patients in the 3-week SMS text message group (aOR 1.25, 95% CI 1.00-1.56) were more likely to complete the screening test. Within the Cologuard-only model, patients in the 3-week SMS text message group (aOR 7.01, 95% CI 1.96-25.07) and the 6-week SMS text message group (aOR 5.75, 95% CI 1.53-21.61) had significantly higher odds of CRC screening completion.

**Conclusions:**

The findings highlight that moderate-frequency SMS text messaging reminders can effectively increase CRC screening rates in FQHCs; however, critical factors include the timing and frequency of these reminders. The 3-week intervention was associated with improved screening uptake, whereas the 6-week theory-informed intervention did not demonstrate a significant advantage over the control group, potentially reflecting a ceiling effect or message fatigue associated with more frequent messaging. Additionally, the study highlights unique screening patterns that contradict previous literature, underscoring the importance of a tailored approach for vulnerable communities.

## Introduction

Colorectal cancer (CRC) is a growing public health concern in the United States, with an estimated 152,810 new cases and 53,010 deaths in 2024 [1]. As a leading cause of cancer-related deaths in the United States [1], professional organizations, including the American Cancer Society, have emphasized the importance of increasing CRC screening awareness and uptake. While the US Preventive Services Task Force typically recommends screening for CRC in all adults aged 50 to 75 years, recent US Preventive Services Task Force recommendations now suggest earlier screening starting at the age of 45 years [2] due to the rising incidence of CRC in individuals aged under 50 years [3].

Recent studies suggest that 71.8% of adults aged 50 to 75 years were up to date with CRC screening, reflecting a modest increase from previous years [4]. Despite this progress, screening disparities persist in underserved communities [5], especially among patients served by federally qualified health centers (FQHCs). FQHCs serve as a vital safety net in the US health care system, providing care to over 50 million individuals annually [6,7]. These centers predominantly serve populations that are economically and socially vulnerable. Recent estimates suggest that over 90% of patients seen at FQHCs reported household incomes below 200% of the federal poverty threshold, with nearly half enrolled in Medicaid and one-quarter uninsured. While FQHCs provide CRC screenings, utilization rates have been historically low, with work by Amboree et al [8] reporting an average screening rate of 40.2% among eligible individuals in FQHCs compared to 72.3% among the general US population. As such, CRC continues to disproportionately affect underserved populations in the United States. Among both men and women, American Indian or Alaska Native and non-Hispanic Black populations experience the highest rates of CRC incidence and mortality [9]. Notably, Black individuals experience 32% more CRC-related deaths compared to White individuals [10].

In efforts to increase screening rates, SMS text messaging reminders have become common. SMS text messaging reminders have proven effective across various health care settings, with a review by Schwebel and Larimer [11] finding that the majority of SMS text messaging reminder studies have shown improved patient appointment and medical compliance. The literature on SMS reminders for CRC screening in general settings currently suggests a limited impact. For example, Uy et al [12] in their systematic review, reported modest increases in breast and cervical cancer screening completion following SMS text messaging interventions, but only small effects for CRC screening. Considering SMS text messaging reminders have been shown to significantly boost screening rates for other cancers [12,13], the research team hypothesized that with theory of behavior change (TOBC)–informed SMS text messages that incorporate behavioral nudges, personalized messaging, and actionable prompts to reduce structural and motivational barriers may be better suited to improve CRC screening uptake in FQHC settings.

Recent evidence suggests a role for infusing established TOBC in SMS text messages [14-16]. Specifically, constructs from the health belief model [17] and the theory of planned behavior [18] emphasize key determinants of behavior that can potentially increase patient motivation, enhance confidence in completing recommended screenings, and provide cues to take actionable steps toward completing their cancer screening. This study examines the effectiveness of a TOBC-informed SMS intervention on CRC screening completion among overdue patients in 2 large FQHC networks in Texas and California.

## Methods

### Sample

**Figure 1.**
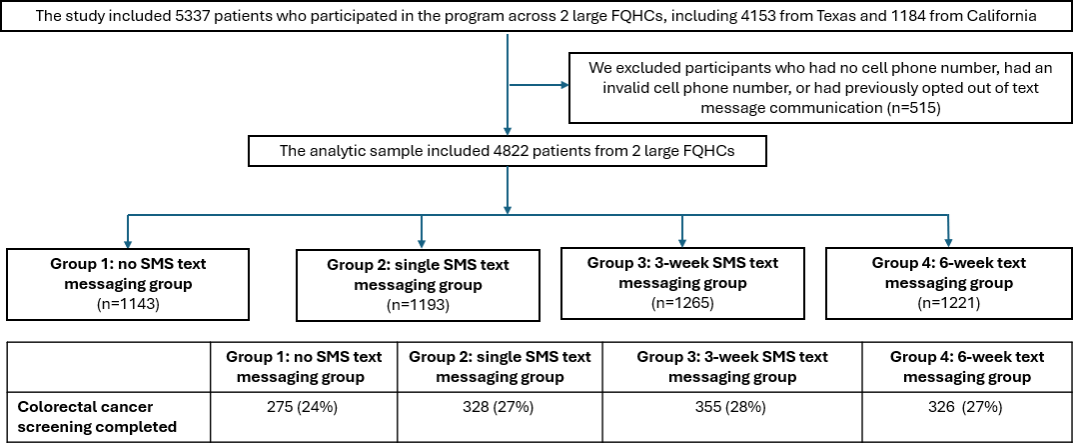
Flowchart of the colorectal cancer test data collection process: (1) group 1: control group, (2) group 2: single message group, (3) group 3: three-week message group, and (4) group 4: six-week message group. FQHC: federally qualified health centers.

The study sample built on prior quality improvement work [19], which enrolled 5337 patients who were overdue for CRC screening from 2 FQHCs: site 1 (56 clinics, Texas) and site 2 (2 full-time and 5 satellite clinics, California). Data were collected from May 2023 to July 2024. A total of 5337 patients were assigned to 1 of 4 study groups over a 90-day period. These included (1) control (no SMS text messages), (2) a single overdue reminder message, (3) a 3-week reminder schedule (1 message per week; total of 3 messages), and (4) a 6-week theory-informed messaging schedule (3 messages per week; total of 18 messages). The unit of randomization (ie, the level at which the intervention was assigned) was the patient, and participants were assigned in a 1:1:1:1 ratio to 1 of the 4 groups using a computer-generated randomization list. After excluding 515 (10%) participants who had no phone number, had an invalid mobile phone number, or had previously opted out of SMS text messaging communication, the final analytic sample consisted of 4822 participants: 1143 in the control group, 1193 in the single message group, 1265 in the 3-week message group, and 1221 in the 6-week SMS text message group (Figure 1).

### Ethical Considerations

This study was reviewed and approved by the institutional review board of the University of Houston (STUDY00004081). All procedures were conducted in accordance with relevant guidelines and regulations. In this study, SMS text messaging reminders were implemented as part of an ongoing quality improvement initiative. As such, patient consent beyond the usual SMS text messaging opt-out procedures was not required. Patient privacy was protected through the use of deidentified data for analysis, and all data linkage between the electronic health record system and the SMS text messaging vendor platform was conducted in accordance with institutional data governance and HIPAA (Health Insurance Portability and Accountability Act)-compliant procedures.

### Quality Improvement Intervention

In 2023, the FQHCs implemented an SMS text messaging reminder initiative to increase cancer screening uptake among individuals overdue for their screenings. After identifying eligible patients, the FQHC worked with academic partners to randomly assign eligible patients into 1 of 4 study groups described above. In partnership with an SMS text messaging reminder service, the FQHC sent messages to patients in the 3 interventions. Patients in group 4 were the only participants who received TOBC-informed SMS text messages. These were designed to be bidirectional SMS text messages, allowing the recipient to respond either to opt out of the message program, answer a question, or respond to a survey. Messages were delivered in both English and Spanish, and the language was matched to each patient’s documented language preference. Examples of TOBC messages include “The overall rates of colon cancer have gone down as more people are getting regular screening. But colon cancer in people younger than age 50 is going up. Because of this, it is now recommended that everyone have their first screen at age 45. Watch this: https://msg.care/8Snk4. Call to get tested: ###-###-####”; “Prioritize your health and get cancer screenings done to make sure you will be there to take care of the ones you love.”; “Listen as Terrance Howard talks about losing his mom to colon cancer: https://msg.care/8Snkw.”

### Measures

The outcome of interest was a binary measure indicating whether participants completed a CRC screening test (fecal immunochemical test [FIT], colonoscopy, or Cologuard) within 90 days of the intervention (0=no, 1=yes). Overall, 87.27% (n=4208) were in the FIT group, while 5.89% (n=284) were in the colonoscopy group and 6.84% (n=330) were in the Cologuard group. The independent variables included demographic characteristics including age, race or ethnicity, primary language, and insurance status. Other independent variables included health conditions—heart disease, diabetes mellitus, chronic lower respiratory diseases, overweight or obesity, hypertension, depression, and mood disorders.

On the basis of the patient’s zip code, we included the following city- and county-level independent variables: (1) 2023 city population, (2) county-level population to primary care physician (PCP) ratio, and (3) city-level CRC death rate. The city population was based on the population estimate as of April 1, 2020, from the US census. The county-level population to PCP ratio was sourced from the County Health Rankings & Roadmaps website [20]. The city-level CRC death rate was sourced from the City Health Dashboard website [21].

### Statistical Analysis

Statistical descriptions were conducted to describe patients and their demographic and health status. Four multiple logistic regression models were used to determine the relationship between independent variables and whether the participant underwent a CRC screening test within 90 days of the intervention. The primary regression model included all participants across all 3 CRC screening test interventions. Subsequently, 3 additional regression models were estimated separately for participants in each screening subgroup: FIT, Cologuard, and colonoscopy. Variance inflation factor (VIF) was used to rule out multicollinearity. All analyses were conducted using R (version 4.4.2; R Foundation for Statistical Computing). Statistical significance was defined as *P*<.05, and results are reported as adjusted odds ratios (aORs), SEs, and 95% CIs.

## Results

Table 1 summarizes the descriptive statistics for CRC screening completion, and Figure 1 illustrates the distribution of participant response messages. In the control group, 24.06% (n=275) of participants completed the screening test, compared to 27.49% (n=328) in the single message group. Completion rates were 28.06% (n=355) in cohort 3 (3-week message group) and 26.7% (n=326) in cohort 4 (6-week message group).

**Table 1.** Patient characteristics and results of the primary regression model (N=4822).

Variable category	Frequency, n (%)	aOR^a^ (SE)	*P* value
Study group			
Control group	1143 (23.70)	R^b^ef	—^c^
Single message	1193 (24.74)	1.22 (0.10)	.04
3-week messages	1265 (26.23)	1.27 (0.10)	.02
6-week messages	1221 (25.32)	1.15 (0.10)	.14
Age group (years)			
45-59	2554 (52.97)	Ref	—
60-64	1078 (22.35)	0.53 (0.09)	<.001
65-69	781 (16.20)	0.52 (0.11)	<.001
>70	409 (8.48)	0.47 (0.15)	<.001
Sex			
Male	1998 (41.44)	Ref	—
Female	2824 (58.56)	1.10 (0.07)	.18
Race or ethnicity			
Asian	197 (4.09)	0.89 (0.20)	.54
Black or African American	622 (12.90)	1.02 (0.13)	.88
Non-Hispanic White	883 (18.31)	Ref	—
Other or unknown	397 (8.23)	1.05 (0.16)	.76
Hispanic	2813 (58.34)	0.98 (0.07)	.84
Primary language			
English	2520 (52.26)	Ref	—
Spanish	2016 (41.81)	1.20 (0.09)	.04
Other	286 (5.93)	1.33 (0.15)	.06
Insurance type			
Uninsured	2178 (45.17)	1.04 (0.12)	.75
Medicaid	1046 (21.69)	Ref	—
Medicare	692 (14.35)	1.24 (0.14)	.12
Private	906 (18.79)	1.10 (0.13)	.46
Diabetes			
No	3332 (69.10)	Ref	—
Yes	1490 (30.90)	0.83 (0.08)	.02
Depression			
No	3580 (74.24)	Ref	—
Yes	1242 (25.76)	0.86 (0.08)	.07
Hypertension			
No	1979 (41.04)	Ref	—
Yes	2843 (58.96)	1.06 (0.13)	.13
Obesity or overweight			
No	934 (19.37)	Ref	—
Yes	3888 (80.63)	1.39 (0.10)	<.001
Heart disease			
No	2355 (48.84)	Ref	—
Yes	2467 (51.16)	0.65 (0.14)	.003
US state			
California	1136 (23.56)	1.32 (0.26)	.29
Texas	3686 (76.44)	Ref	—
Chronic lower respiratory diseases 2022			
No	4478 (92.87)	Ref	—
Yes	344 (7.13)	0.93 (0.15)	.63
2021 city-level colorectal cancer death rate^d^			
Less than the average	4122 (85.48)	Ref	—
Higher than the average	700 (14.52)	1.11 (0.20)	.53
2023 city size^e^			
Small town or city (<150,000)	577 (11.97)	Ref	—
Large city (>150,000)	4245 (88.03)	1.19 (0.27)	.66
2021 county-level population to PCP^f^ ratio^g^			
Less than the average	1637 (33.95)	Ref	—
Higher than the average	3185 (66.05)	1.17 (0.25)	.63

a aOR: adjusted odds ratio.

b REF: reference group.

c Not applicable.

d The 2021 city-level colorectal cancer death rate is based on the City Health Dashboard website. The average city-level colorectal cancer death ratio is calculated by summing the colorectal cancer death rates of all sample cities and dividing by the number of cities in the sample.

e The city population is based on the population estimate as of April 1, 2020 (V2023), from the US census.

f PCP: primary care physician.

g The 2021 county-level population to primary care physician (PCP) ratio is sourced from the County Health Ranking & Roadmaps website. The average county-level population to PCP ratio is calculated by summing the population to PCP rates of all sample cities and dividing by the number of cities in the sample.

Table 1 shows the characteristics and combined 3-test regression results. Overall, 23.7% (n=1143) were in the control group, 24.74% (n=1193) in the single message group, 26.23% (n=1265) in the 3-message group, and 25.32% (n=1221) in the 6-week message group. The age distribution showed that the majority of patients were aged 45 to 59 years (n=2554, 52.97%), followed by those aged 60 to 64 years (n=1078, 22.35%), 65 to 69 years (n=781, 16.2%), and ≥70 years (n=409, 8.48%). Most patients were female (n=2824, 58.56%). The racial composition was as follows: 58.34% (n=2813) Hispanic, 18.31% (n=883) White, 8.23% (n=397) in other or unknown racial categories, 12.90% (n=622) Black or African American, and 4.09% (n=197) Asian. Most patients reported English as their primary language (n=2520, 52.26%), followed by Spanish (n=2016, 42.81%), and other languages (n=286, 5.93%). Most patients were from Texas (n=3686, 76.44%), and the remaining patients were from California (n=1136, 23.56%). Regarding insurance, 45.17% (n=2178) were uninsured, 18.79% (n=906) had private insurance, 14.35% (n=692) had Medicaid, and 21.69% (n=1046) had Medicare. Additionally, 80.63% (n=3888) were obese or overweight, 30.9% (n=1490) had diabetes, 7.13% (n=344) had chronic lower respiratory disease, 51.16% (n=2467) had heart disease, 58.96% (n=2843) had hypertension, and 25.76% (n=1242) had a history of depression or mood disorders. At the community level, 85.48% (n=4122) of patients lived in cities where the CRC death rate was below the sample average, and most patients (n=4245, 88.03%) were from large cities with populations of ≥150,000. Additionally, 66.05% (n=3185) lived in cities where the county-level PCP ratio was at or above the sample average.

The VIF test showed that the combined 3-test model, the FIT model, and the Cologuard model did not exhibit evidence of multicollinearity. Table 1 shows that patients in the single message group (aOR 1.22, 95% CI 1.00-1.47) and 3-week message group (aOR 1.27, 95% CI 1.05‐1.53) were more likely to complete the screening test. Compared to patients aged 45 to 59 years, those aged 60 to 65 years (aOR 0.53, 95% CI 0.45‐0.64), 65 to 69 years (aOR 0.52, 95% CI 0.42‐0.55), and >70 years (aOR 0.47, 95% CI 0.35‐0.64) were less likely to complete the test. Regarding language, Spanish-speaking patients (aOR 1.20, 95% CI 1.00‐1.43) were more likely to complete the test. Regarding health conditions, patients with diabetes (aOR 0.83, 95% CI 0.70‐0.97) were more likely to complete the test, whereas patients with obesity or overweight (aOR 1.39, 95% CI 1.15‐1.68) were more likely to complete the test. In contrast, those with heart disease (aOR 0.65, 95% CI 0.49‐0.86) were less likely to complete it.

Tables2  3 present the results for the FIT, colonoscopy, and Cologuard tests. For FIT (Table 2), the regression model shows that patients in the 3-message group (aOR 1.25, 95% CI 1.00‐1.56) were more likely to complete the screening test. Compared to patients aged 45 to 59 years, those aged 60 to 65 years (aOR 0.64, 95% CI 0.53‐0.79), 65 to 69 years (aOR 0.59, 95% CI 0.45‐0.77), and >70 years (aOR 0.48, 95% CI 0.33‐0.69) were less likely to complete the test. In terms of language, Spanish-speaking patients (aOR 1.54, 95% CI 1.24‐1.91) and speakers of other languages (aOR 1.55, 95% CI 1.12‐2.15) were more likely to complete the test. Compared to patients with Medicaid, those without any insurance (aOR 0.58, 95% CI 0.44‐0.77), with Medicare (aOR 0.56, 95% CI 0.40‐0.79), and with private insurance (aOR 0.30, 95% CI 0.22‐0.40) were more likely to complete it. Patients with hypertension (aOR 0.72, 95% CI 0.60‐0.87) and those with obesity or overweight (aOR 0.73, 95% CI 0.60‐0.87) were less likely to complete the test. Patients living in cities with higher CRC death rates (aOR 0.53, 95% CI 0.31‐0.90) were also less likely to complete it. Finally, patients residing in California (aOR 2.51, 95% CI 1.44‐4.40) were more likely to complete it compared to those living in Texas.

**Table 2.** Colorectal cancer results of the fecal immunochemical test–only and colonoscopy–only models.

Variable category	FIT^b^ (n=4208)				Colonoscopy (n=284)			
	Frequency, n (%)	aOR^c^ (SE)	*P* value	95% CI	Frequency, n (%)	aOR (SE)	*P* value	95% CI
Study group								
Control group	1012 (24.05)	R^d^ef	—^e^	—	61 (21.48)	Ref	—	—
Single message	1039 (24.69)	1.14 (0.11)	.26	0.91-1.42	69 (24.30)	1.57 (0.43)	.3	0.67-3.64
3-week messages	1094 (26.00)	1.25 (0.11)	.04	1.00-1.56	79 (27.82)	1.23 (0.42)	.61	0.54-2.80
6-week messages	1063 (25.26)	1.08 (0.11)	.52	0.86-1.34	75 (26.41)	0.95 (0.41)	.91	0.42-2.13
Age group (years)								
45-59	2215 (52.64)	Ref	—	—	121 (42.61)	Ref	—	—
60-64	976 (23.19)	0.64 (0.10)	<.001	0.53-0.79	64 (22.54)	0.61 (0.38)	.19	0.29-1.29
65-69	666 (15.83)	0.59 (0.14)	<.001	0.45-0.77	67 (23.59)	0.55 (0.45)	.18	0.23-1.32
>70	351 (8.34)	0.48 (0.19)	<.001	0.33-0.69	32 (11.27)	0.69 (0.54)	.5	0.24-2.01
Gender								
Male	1178 (42.25)	Ref	—	—	102 (35.92)	Ref	—	—
Female	2430 (57.75)	1.07 (0.08)	.41	0.91-1.26	182 (64.08)	1.03 (0.30)	.91	0.57-1.87
Race or ethnicity								
Asian	172 (4.09)	0.76 (0.27)	.3	0.45-1.28	9 (3.17)	3.42 (0.83)	.14	0.67-17.17
Black or African American	530 (12.60)	1.11 (0.16)	.53	0.80-1.53	50 (17.61)	1.16 (0.46)	.76	0.47-2.86
Non-Hispanic White	717 (17.04)	Ref	—	—	71 (25.00)	Ref	—	—
Other or unknown	259 (6.15)	0.95 (0.21)	.82	0.63-1.44	21 (7.39)	1.09 (0.60)	.89	0.34-3.49
Hispanic	2530 (60.12)	1.07 (0.14)	.64	0.81-1.41	133 (46.83)	0.95 (0.42)	.9	0.42-2.16
Primary language								
English	2087 (49.60)	Ref	—	—	192 (67.61)	Ref	—	—
Spanish	1849 (43.94)	1.54 (0.11)	<.001	1.24-1.91	82 (28.87)	2.48 (0.37)	.02	1.18-5.12
Other	272 (6.46)	1.55 (0.17)	.01	1.12-2.15	10 (3.52)	2.75 (0.82)	.22	0.55-13.71
Insurance type								
Uninsured	2068 (49.14)	Ref	—	—	62 (21.83)	Ref	—	—
Medicaid	947 (22.50)	0.58 (0.14)	<.001	0.44-0.77	56 (19.72)	3.55 (0.52)	.01	1.28-9.83
Medicare	539 (12.81)	0.56 (0.17)	<.001	0.40-0.79	84 (29.58)	1.83 (0.52)	.24	0.65-5.11
Private	654 (15.54)	0.30 (0.16)	<.001	0.22-0.40	82 (28.87)	3.11 (0.50)	.02	1.16-8.32
Hypertension								
No	1187 (28.21)	Ref	—	—	85 (29.93)	Ref	—	—
Yes	3021 (71.79)	0.72 (0.09)	<.001	0.60-0.87	199 (70.07)	0.81 (0.32)	.51	0.43-1.52
Obesity or overweight								
No	341 (8.10)	Ref	—	—	21 (7.39)	Ref	—	—
Yes	3867 (91.90)	0.73 (0.15)	.03	0.54-0.97	263 (92.71)	0.65 (0.54)	.42	0.43-1.52
2021 city-level cancer death rate								
Less than average	3603 (85.62)	Ref	—	—	247 (86.97)	Ref	—	—
Higher than average	605 (14.38)	0.53 (0.28)	.02	0.31-0.90	37 (13.03)	0.60 (0.75)	.49	0.14-2.69
2023 city size								
Small town or city (<150,000)	516 (12.26)	Ref	—	—	65 (24.89)	Ref	—	—
Large city (≥150,000)	3692 (87.74)	0.77 (0.33)	.42	0.40-1.46	219 (77.11)	0.40 (1.18)	.27	0.03-3.36
US state								
California	1094 (26.00)	Ref	—	—	42 (17.36)	Ref	—	—
Texas	3114 (74.00)	2.51 (0.29)	<.001	1.44-4.40	242 (86.64)	2.67 (1.15)	.04	0.35-32.28
2021 county-level								
Population to PCP^f^ ratio								
Less than the average	1535 (36.48)	Ref	—	—	65 (22.89)	Ref	—	—
Higher than the average	2673 (63.52)	0.85 (0.28)	.56	0.49-1.48	219 (77.11)	1.28 (1.17)	.23	0.13-12.90

b FIT: fecal immunochemical test.

c aOR: adjusted odds ratio.

d Ref: reference group.

e Not applicable.

f PCP: primary care physician.

**Table 3.** Colorectal cancer results of the Cologuard-only model (n=330).

Variable category	Frequency, n (%)	aOR^a^ (SE)	*P* value	95% CI
Study group				
Control group	70 (21.21)	R^b^ef	—^c^	—
Single message	85 (25.76)	3.20 (0.60)	.05	0.98-10.37
3-week messages	92 (27.88)	7.01 (0.64)	<.001	1.96-25.07
6-week messages	93 (25.15)	5.75 (0.68)	.01	1.53-21.61
Age group (years)				
45-59	218 (66.06)	Ref	—	—
60-64	38 (11.52)	0.31 (0.82)	.27	0.50-12.38
65-69	48 (14.55)	0.30 (0.89)	.78	0.14-4.47
>70	26 (7.88)	0.40 (0.82)	.7	0.15-3.64
Gender				
Male	118 (35.76)	Ref	—	—
Female	212 (64.26)	1.17 (0.53)	.76	0.42-3.28
Race				
Asian or Black or African American or others or unknown	95 (28.79)	21.30 (1.15)	.01	2.26-201.09
Non-Hispanic White	95 (28.79)	Ref	—	—
Hispanic	140 (42.42)	1.10 (0.53)	.86	0.39-3.11
Primary language				
English	242 (73.33)	Ref	—	—
Spanish or other	88 (26.67)	2.07 (0.63)	.25	0.61-7.08
Insurance type				
Uninsured	47 (14.24)	1.32 (0.99)	.78	0.19-9.20
Medicaid	43 (13.03)	Ref	—	—
Medicare	70 (21.21)	0.86 (0.78)	.84	0.18-3.98
Private	170 (51.52)	0.62 (0.72)	.51	0.15-2.54
Diabetes				
No	223 (67.58)	Ref	—	—
Yes	107 (32.42)	1.68 (0.59)	.38	0.53-5.30
Depression				
No	241(73.03)	Ref	—	—
Yes	89 (26.97)	1.48 (0.62)	.52	0.44-4.97
Hypertension				
No	140 (42.42)	Ref	—	—
Yes	190 (57.58)	0.56 (0.58)	.31	0.61-7.08
Obesity or overweight				
No	41 (12.42)	Ref	—	—
Yes	289 (87.58)	0.95 (0.68)	.93	0.25-3.57
Chronic lower respiratory diseases				
No	304 (91.52)	Ref	—	—
Yes	26 (7.88)	3.71 (1.12)	.24	0.41-33.14
Family has any cancer				
No	302 (92.12)	Ref	—	—
Yes	28 (8.48)	1.14 (0.96)	.89	0.17-7.48
Visit count in 2022				
0	87 (26.36)	Ref	—	—
1-2	72 (21.82)	2.09 (0.77)	.34	0.46-9.46
3-4	65 (19.70)	1.46 (0.80)	.64	0.31-6.93
>5	106 (32.12)	1.07 (0.76)	.93	0.24-4.71
2022 annual wellness visit				
No	254 (76.97)	Ref	—	—
Yes	76 (23.03)	0.55 (0.61)	.33	0.17-4.97
City-level colorectal cancer death rate				
Less than average	272 (82.42)	Ref	—	—
Higher than average	58 (17.58)	1.62 (1.18)	.69	0.16-16.44
County-level population to PCP^d^ ratio				
Less than the average	37 (11.21)	Ref	—	—
Higher than the average	293 (88.79)	1.25 (1.34)	.87	0.09-17.23

a aOR: adjusted odds ratio.

b REF: reference group.

c Not applicable.

d PCP: primary care physician.

For the colonoscopy test (Table 2), compared to English-speaking patients, Spanish-speaking patients (aOR 2.48, 95% CI 1.18‐5.12) were more likely to complete the test. Compared to patients with Medicaid, uninsured patients (aOR 3.55, 95% CI 1.28‐9.83) and those with private insurance (aOR 3.11, 95% CI 1.16‐8.32) were more likely to complete the test. For the Cologuard model (Table 3), the city size variable was removed from the analysis because its VIF exceeded 5, indicating potential multicollinearity. Additionally, for categorical variables with very small cell counts in certain categories, we combined categories to avoid sparse data issues and improve the robustness of the estimates. Compared to the control group, the intervention 3-week message group (aOR 7.01, 95% CI 1.96‐25.07) and 6-week message group (aOR 5.75, 95% CI 1.53‐21.61) were more likely to complete the test. Compared to White, other races (Asian, Black or African American, others, and unknown; aOR 21.30, 95% CI 2.26‐201.09) were more likely to complete it. In the Cologuard model, we observed wide 95% CIs for several variables, indicating imprecision in the estimates. In particular, the race or ethnicity estimate was 21.30 with a correspondingly wide CI. To investigate this, we cross-tabulated race or ethnicity by Cologuard test completion and found that among Asian patients (n=95), 94 (99%) completed the test. This near-complete separation (ie, very high completion within a subgroup) likely contributed to the inflated estimate and wide 95% CI.

## Discussion

### Principal Findings

In this study of TOBC-informed SMS text messaging reminders for CRC screening in an FQHC, the combined 3-test model, FIT model, and Cologuard model showed that sending 1 reminder per week for 3 weeks significantly increased screening rates. This aligns with existing work on the overall impact of SMS text messaging reminder interventions, with meta-analyses associating SMS text messaging reminders with improved general appointment and medical compliance [11]. However, results for screening settings vary, with work finding some [22-24] and no changes [5,25,26] following SMS text messaging reminders. Our findings on reminder frequency align with previous research, showing that 2 reminders are more effective than a single reminder in reducing missed appointments and same-day cancelations [27]. Additionally, work by Teo et al [28] suggests that excessive reminders can cause “reminder fatigue,” leading to redundancy and inattention, aligning with the no effect seen in the 6 weeks messaging group.

Age was found to be a significant factor, with screening rates being inversely associated with advancing age. This finding aligns with Walter et al [29], who found screening rates to decrease with increasing age in a Veterans Affairs clinic setting. While current guidelines recommend a colonoscopy every 10 years for adults aged 45 years and older [30], Patel et al [31] emphasized that screening decisions for older adults should be individualized based on prior screening history and patient preference. However, given the heightened challenges older adults face in accessing care [32], this finding raises concern that, in practice, older adults may not receive appropriate follow-up or adhere to these tailored recommendations. Obesity or overweight was another significant predictor, as patients who identified as having obesity were associated with greater CRC screening rates compared to patients without obesity. Prior work on BMI status and screening rates has yielded mixed findings, with obesity reported as both a negative [33,34] and positive predictor for screening [35]. Notably, Kendall et al [36] found obesity status to be a positive predictor for screening among Medicare beneficiaries. Patients in FQHC settings who receive more frequent care for obesity management have increased opportunities to be assessed and recommended for screening.

Our findings also highlighted that patients with diabetes had lower CRC screening uptake. These findings are consistent with prior studies demonstrating a negative association between diabetes and CRC screening [37,38]. We believe that patients with diabetes often have multiple comorbidities, which may contribute to competing health demands and reduce the likelihood of completing preventive screening. Similarly, patients with heart disease were associated with decreased CRC screening uptake compared to patients without heart disease. This finding is consistent with Bhatia et al [39], who also found lower screening rates among patients with heart failure and cardiovascular disease, citing competing demands of more intensive chronic disease management over preventative care. However, because unfavorable cardiovascular risk factors are linked to an increased risk of CRC [40], a critical need exists to enhance access to preventive screening services for patients managing cardiac comorbidities within FQHC settings.

On the basis of the results of FIT and colonoscopy models, patients who primarily spoke Spanish or other languages were more likely to complete CRC screening, compared to patients who were English speakers. This finding is unique among current literature, as work suggests that patients with limited English-language proficiency are less likely to receive physician recommendations for a colonoscopy and have lower screening rates [41,42]. As FQHCs serve a large non-English-speaking patient population, this highlights their ability to increase awareness of and access to preventive care across the minoritized populations they serve. Additionally, FQHCs often provide culturally tailored education and language-concordant care, which can enhance trust and communication between patients and providers [43,44]. This supportive environment helps non-English-speaking patients understand the screening process and feel comfortable completing tests, thereby boosting screening rates despite language barriers.

Uninsured patients were associated with greater CRC screening rates compared to patients with Medicaid, while having Medicare and private insurance did not lead to a significant difference. These findings are unique among most work covering general clinic settings, which argues that uninsured patients [45,46] and those without nonprivate insurance are associated with lower adherence to CRC screening guidelines [47]. Given the sliding scale this setting uses [48], our observed screening uptake potentially speaks to the FQHC’s ability to provide free or low-cost preventive service use among those who are uninsured. Notably, the clinics in this study provide a free FIT kit, mailing it with prepaid return envelopes to eliminate the need for clinic visits.

State of residence was a major factor influencing screening rates, with patients in California showing higher screening rates compared to those in Texas. This disparity is particularly pronounced in safety-net settings, as Texas has one of the largest uninsured populations in the nation [49]. Furthermore, CRC screening coverage was only recently included under Medicare in 2021 [50], further complicating access for underserved patients. CRC screening rates at Texas FQHCs remain below the national average, with only 35% of eligible patients up to date on screenings in 2020 [51]. A key contributor to the difference in screening rates between California and Texas is the variation in Medicaid expansion policies [52]. California’s Medicaid expansion has resulted in the majority of adult FQHC patients having some form of insurance coverage, improving access to preventive services [53,54]. Texas, a nonexpansion state, leaves many FQHC patients uninsured. Despite free FIT kits, lack of consistent coverage likely lowers screening engagement, highlighting the impact of state policy on underserved populations.

There are many limitations to be considered in interpreting these findings. First, screening tests completed outside the FQHC systems may not have been captured, potentially leading to underestimation of true screening uptake. Second, the study included only 2 FQHC organizations, which may limit generalizability to non-FQHC settings. Third, the follow-up period was limited to 90 days, preventing assessment of longer-term adherence. Fourth, exact timing information (eg, weekends, holidays, or time of day) was not collected, limiting our ability to assess potential timing effects on participant response. Additionally, some variables, including community-level characteristics, may have been measured with limited precision or at a relatively crude level. Finally, for the colonoscopy and Cologuard models, given the relatively small sample sizes and the large ORs observed for some variables, the results should be interpreted with caution. Future studies should examine the effect of additional optimization variables (eg, message timing and length) to assess effectiveness.

### Conclusions

In an FQHC study, 3 weekly TOBC-informed SMS text messaging reminders most effectively improved CRC screening. Screening rates also varied by age, language, insurance, obesity, heart disease, and state, underscoring the need to further explore TOBC-informed reminders to promote equitable screening.

## Abbreviations

aOR: adjusted odds ratio
CRC: colorectal cancer
FIT: fecal immunochemical test
FQHC: federally qualified health center
HIPAA: Health Insurance Portability and Accountability Act
TOBC: theories of behavior change
VIF: variance inflation factor

## References

[1] Siegel RL, Miller KD, Fuchs HE, Jemal A (2021). Cancer statistics, 2021. CA Cancer J Clin.

[2] Davidson KW, Barry MJ, US Preventive Services Task Force (2021). Screening for colorectal cancer: US Preventive Services Task Force recommendation statement. JAMA.

[3] Siegel RL, Miller KD, Goding Sauer A (2020). Colorectal cancer statistics, 2020. CA Cancer J Clin.

[4] Ebner DW, Finney Rutten LJ, Miller-Wilson LA (2024). Trends in colorectal cancer screening from the National Health Interview Survey: analysis of the impact of different modalities on overall screening rates. Cancer Prev Res (Phila).

[5] Coronado GD, Rivelli JS, Fuoco MJ (2018). Effect of reminding patients to complete fecal immunochemical testing: a comparative effectiveness study of automated and live approaches. J Gen Intern Med.

[6] America’s health centers: by the numbers. NACHC.

[7] Masselil M, Sin P (2025). How many patients do community health centers really care for? You’ll be surprised.

[8] Amboree TL, Montealegre JR, Parker SL (2024). National breast, cervical, and colorectal cancer screening use in federally qualified health centers. JAMA Intern Med.

[9] Siegel RL, Wagle NS, Cercek A, Smith RA, Jemal A (2023). Colorectal cancer statistics, 2023. CA Cancer J Clin.

[10] Anyane-Yeboa A, Bermudez H, Fredericks M (2025). The revised colorectal cancer screening guideline and screening burden at community health centers. Sci Rep.

[11] Schwebel FJ, Larimer ME (2018). Using text message reminders in health care services: a narrative literature review. Internet Interv.

[12] Uy C, Lopez J, Trinh-Shevrin C, Kwon SC, Sherman SE, Liang PS (2017). Text messaging interventions on cancer screening rates: a systematic review. J Med Internet Res.

[13] Kerrison RS, Shukla H, Cunningham D, Oyebode O, Friedman E (2015). Text-message reminders increase uptake of routine breast screening appointments: a randomised controlled trial in a hard-to-reach population. Br J Cancer.

[14] Sharma V, Feldman M, Sharma R (2024). Telehealth technologies in diabetes self-management and education. J Diabetes Sci Technol.

[15] Fishbein M, Hennessy M, Kamb M (2001). Using intervention theory to model factors influencing behavior change. Project RESPECT. Eval Health Prof.

[16] Berset AE, Epstein JN, Hommel KA, Brinkman WB (2023). Examining the unified theory of behavior change constructs among adolescents taking attention-deficit/hyperactivity disorder medicine: a longitudinal study. Acad Pediatr.

[17] Alyafei A, Easton-Carr R (2026). StatPearls [Internet].

[18] Conner M, Tenenbaum G, Eklund RC (2020). Handbook of Sport Psychology.

[19] Xu T, Chavez S, Angelocci T, Adepoju O (2025). Does text messaging increase breast cancer screening gap closure rates?: evidence from federally qualified health centers. JMIR Mhealth Uhealth.

[20] Kingery KL (2018). County health rankings & roadmaps. J Youth Dev.

[21] Gourevitch MN, Athens JK, Levine SE, Kleiman N, Thorpe LE (2019). City-level measures of health, health determinants, and equity to foster population health improvement: the City Health Dashboard. Am J Public Health.

[22] Muller CJ, Robinson RF, Smith JJ (2017). Text message reminders increased colorectal cancer screening in a randomized trial with Alaska Native and American Indian people. Cancer.

[23] Weaver KE, Ellis SD, Denizard-Thompson N, Kronner D, Miller DP (2015). Crafting appealing text messages to encourage colorectal cancer screening test completion: a qualitative study. JMIR Mhealth Uhealth.

[24] Vives N, Travier N, Farre A (2024). Effectiveness and acceptability of targeted text message reminders in colorectal cancer screening: randomized controlled trial (M-TICS study). JMIR Public Health Surveill.

[25] Vives N, Binefa G, Travier N (2025). Text messaging versus postal reminders to improve participation in a colorectal cancer screening program: randomized controlled trial. JMIR Mhealth Uhealth.

[26] Hamad W, Purushotham A, Hughes S, Round T (2024). Text-message reminders to increase participation in colorectal cancer screening. Br J Gen Pract.

[27] Ulloa-Pérez E, Blasi PR, Westbrook EO, Lozano P, Coleman KF, Coley RY (2022). Pragmatic randomized study of targeted text message reminders to reduce missed clinic visits. Perm J.

[28] Teo AR, Metcalf EE, Strange W (2021). Enhancing usability of appointment reminders: qualitative interviews of patients receiving care in the Veterans Health Administration. J Gen Intern Med.

[29] Walter LC, Lindquist K, Nugent S (2009). Impact of age and comorbidity on colorectal cancer screening among older veterans. Ann Intern Med.

[30] Inadomi JM, Vijan S, Janz NK (2012). Adherence to colorectal cancer screening: a randomized clinical trial of competing strategies. Arch Intern Med.

[31] Patel SG, May FP, Anderson JC (2022). Updates on age to start and stop colorectal cancer screening: recommendations from the U.S. Multi-Society Task Force on colorectal cancer. Gastroenterology.

[32] Fitzpatrick AL, Powe NR, Cooper LS, Ives DG, Robbins JA (2004). Barriers to health care access among the elderly and who perceives them. Am J Public Health.

[33] Heo M, Allison DB, Fontaine KR (2004). Overweight, obesity, and colorectal cancer screening: disparity between men and women. BMC Public Health.

[34] Rosen AB, Schneider EC (2004). Colorectal cancer screening disparities related to obesity and gender. J Gen Intern Med.

[35] Chang VW, Asch DA, Werner RM (2010). Quality of care among obese patients. JAMA.

[36] Kendall KA, Lee E, Zuckerman IH (2013). Obesity status and colorectal cancer screening in the United States. J Obes.

[37] von Wagner C, Cadar D, Hackett RA (2020). Type 2 diabetes and colorectal cancer screening: findings from the English Longitudinal Study of Ageing. J Med Screen.

[38] Wilkinson JE, Culpepper L (2011). Associations between colorectal cancer screening and glycemic control in people with diabetes, Boston, Massachusetts, 2005-2010. Prev Chronic Dis.

[39] Bhatia D, Sutradhar R, Tinmouth J, Singh S, Lau C, Lipscombe LL (2021). Influence of chronic comorbidities on periodic colorectal cancer screening participation: a population-based cohort study. Prev Med.

[40] Zhang C, Cheng Y, Luo D (2021). Association between cardiovascular risk factors and colorectal cancer: a systematic review and meta-analysis of prospective cohort studies. EClinicalMedicine.

[41] Castañeda-Avila MA, Tisminetzky M, Oyinbo AG, Lapane K (2024). Racial and ethnic disparities in use of colorectal cancer screening among adults with chronic medical conditions: BRFSS 2012-2020. Prev Chronic Dis.

[42] Cataneo JL, Kim TD, Park JJ, Marecik S, Kochar K (2022). Disparities in screening for colorectal cancer based on limited language proficiency. Am Surg.

[43] Glaser KM, Crabtree-Ide CR, McNulty AD (2024). Improving guideline-recommended colorectal cancer screening in a federally qualified health center (FQHC): implementing a patient navigation and practice facilitation intervention to promote health equity. Int J Environ Res Public Health.

[44] Lin SC, McKinley D, Sripipatana A, Makaroff L (2017). Colorectal cancer screening at US community health centers: examination of sociodemographic disparities and association with patient-provider communication. Cancer.

[45] Bayly JE, Schonberg MA, Castro MC, Mukamal KJ (2024). Individual and geospatial factors associated with receipt of colorectal cancer screening: a state-wide mixed-level analysis. Fam Med Community Health.

[46] Freund KM, Reisinger SA, LeClair AM (2019). Insurance stability and cancer screening behaviors. Health Equity.

[47] (2018). WHO housing and health guidelines. World Health Organization.

[48] Conway SJ, Murphy J, Efron JE (2024). academic medical centers and federally qualified health centers: collaboration for the care of underserved communities. J Prim Care Community Health.

[49] Olmstead T, Spencer JC, Kluz N, Zhan FB, Shokar NK, Pignone M (2024). Costs and projected effect of a federally qualified health center-based mailed colorectal cancer screening program in Texas. Prev Chronic Dis.

[50] (2021). Abbott takes final step to ensure affordable colorectal cancer screenings for Texans. American Cancer Society Cancer Action Network.

[51] Texas Health Center Program Uniform Data System (UDS) data. Health Resources & Services Administration.

[52] (2026). Status of state Medicaid expansion decisions. KFF.

[53] Zerhouni YA, Trinh QD, Lipsitz S (2019). Effect of Medicaid expansion on colorectal cancer screening rates. Dis Colon Rectum.

[54] Ojinnaka CO, Suri Y (2020). Impact of Medicaid expansion on healthcare access among individuals living with chronic diseases. Am J Prev Med.

